# Skin Tone Parameters as Possible Factors in Humectant Moisturizing Effectiveness

**DOI:** 10.7759/cureus.86665

**Published:** 2025-06-24

**Authors:** Harvey N Mayrovitz, Emily Deehan, Marissa Ruppe, Kawaiola Cael Aoki

**Affiliations:** 1 Medical Education, Nova Southeastern University Dr. Kiran C. Patel College of Allopathic Medicine, Davie, USA; 2 Osteopathic Medicine, Nova Southeastern University Dr. Kiran C. Patel College of Osteopathic Medicine, Davie, USA

**Keywords:** dermal water, erythema, gender differences, individual topical angle, melanin, skin color, skin hydration, skin tone, tdc, tissue dielectric constant

## Abstract

Background: Prior work demonstrated differences in basal skin hydration related to skin tone parameter differences that included the melanin index (M), the erythema index (E), and the individual topology angle (ITA). The impact of these parameters on moisturization-induced hydration remains unclear. This study aimed to investigate whether basal skin tone influences moisturizer-related skin hydration.

Materials and methods: M, E, and ITA were measured bilaterally at multiple anterior forearm sites of 30 young adults. The percentage water content (PWC) was determined by measuring the skin's dielectric constant to depths of 0.5 mm (PWC5) and 2.5 mm. PWC measurements were done before and four hours after applying four different humectant moisturizers. Analyses were conducted using the whole group and subgroups divided according to values below and above the median values for M, E, and ITA.

Results: The whole group PWC increased at both depths. At 0.5 mm, it increased from its baseline of 46.4±10.2 to 51.5±9.1 (p < 0.001), a 12.6±11.3% increase. At 2.5 mm, PWC increased from its baseline of 39.1±6.6 to 40.6±6.1 (p < 0.001), a 4.2±5.7% increase. Both subgroups increased at 0.5 mm. However, percentage increases in PWC tended to be greater for subgroups with lower M and E and higher ITA. Considering gender, only males showed a significant positive correlation between the change in PWC5 and ITA (r = 0.584, p = 0.028), whereas females exhibited a uniform, non-skin tone-dependent response.

Conclusions: The findings suggest that lighter skin tones (higher ITA) may be associated with greater moisturizer-induced skin hydration. However, the differential gender responses may also indicate that male-female differences are involved and warrant further study.

## Introduction

Prior work has demonstrated significant differences in skin properties dependent on skin tone parameter values. In 2002, Aramaki and coworkers reported that European women, who had, on average, significantly lower melanin values than Japanese women, also had significantly larger transepidermal water loss (TEWL) values [1]. Women with lower melanin levels also tended to have higher stratum corneum hydration. An extensive study of stratum corneum hydration differences among American women of various ethnicities and presumed associated variations in skin color parameters revealed age-dependent differences among the groups [2]. They found the lowest stratum corneum hydration in the African American and Caucasian groups, who would have had the highest and lowest melanin levels, respectively.

In another study, no significant age-related changes in skin color parameters were observed over multiple decades; however, a significant decrease in epidermal but not dermal hydration was reported [3]. This suggests that measurements of variable skin depths are important when assessing potential differences in hydration status. A study of 200 Indonesian, 100 Vietnamese, and 97 Singaporean women has provided important data on differential skin properties [4]. Calculations based on their forehead skin data indicate a significantly greater melanin index and erythema index for Indonesian women compared to Vietnamese women, with the Indonesian women having a significantly lower skin hydration. Contrastingly, the Singapore women, who also had significantly lower melanin and erythema values than the Indonesian women, showed no significant difference in forehead skin hydration. A study that included many Chinese and Korean women from different geographical areas found that Korean women with a lower melanin index, derived from individual topology angle (ITA) values, tended to have higher hydration levels, as measured on the cheek and forehead [5].

The ITA is a way to characterize skin color using the CIE (International Commission on Illumination) Lab* color space by determining the angle between the skin lightness parameter (L*) and the yellow-blue parameter (b*), resulting in angle values that range from very light (>55°) to dark (<30°) [6,7]. Furthermore, a study of non-pathological redness-prone faces found that skin hydration levels were elevated compared to a control group [8]. However, measurements on over 1000 young adult Chinese women indicated that women with ITA values corresponding to intermediate levels had less skin hydration than those with higher values, indicating lighter skin tones [9]. Contrastingly, data reported for Black and White women indicate no significant difference in skin hydration as measured at the inner arm, despite significant differences in colorimeter-determined skin lightness [10]. Combined male and female Korean facial hydration data indicate a positive but low correlation to skin tone [11]. These various and sometimes divergent findings raise questions about the role of skin color parameters in the ability to hydrate skin.

The specific aim of the present study was to investigate this issue by determining whether levels of melanin, erythema, and skin ITA values influenced the effectiveness of the aggregate of various humectant moisturizers in hydrating skin, as measured by changes in the skin's tissue dielectric constant (TDC). The goal was to determine the overall response to the application of the aggregate of these moisturizers, rather than to evaluate the relative efficacy of one product compared to another. Thus, the objective of this study was to evaluate whether skin tone parameters, specifically melanin index, erythema index, and ITA, influence the skin hydration response to humectant moisturizers, as measured by changes in the TDC at two skin depths.

## Materials and methods

Participants

A convenience sample of 30 young adult medical students participated in this research study after they read and signed a University Institutional Review Board (IRB)-approved informed consent (IRB no. 2023-550). The participants were then scheduled for a day and time for their measurements. The entry requirements to participate in this study were that a potential participant (1) not have significant hair on the anterior forearm, (2) have no known allergy to topical moisturizers, and (3) have no history of a chronic dermatologic condition in the anatomical area of interest.

Measurements

**Figure 1:**
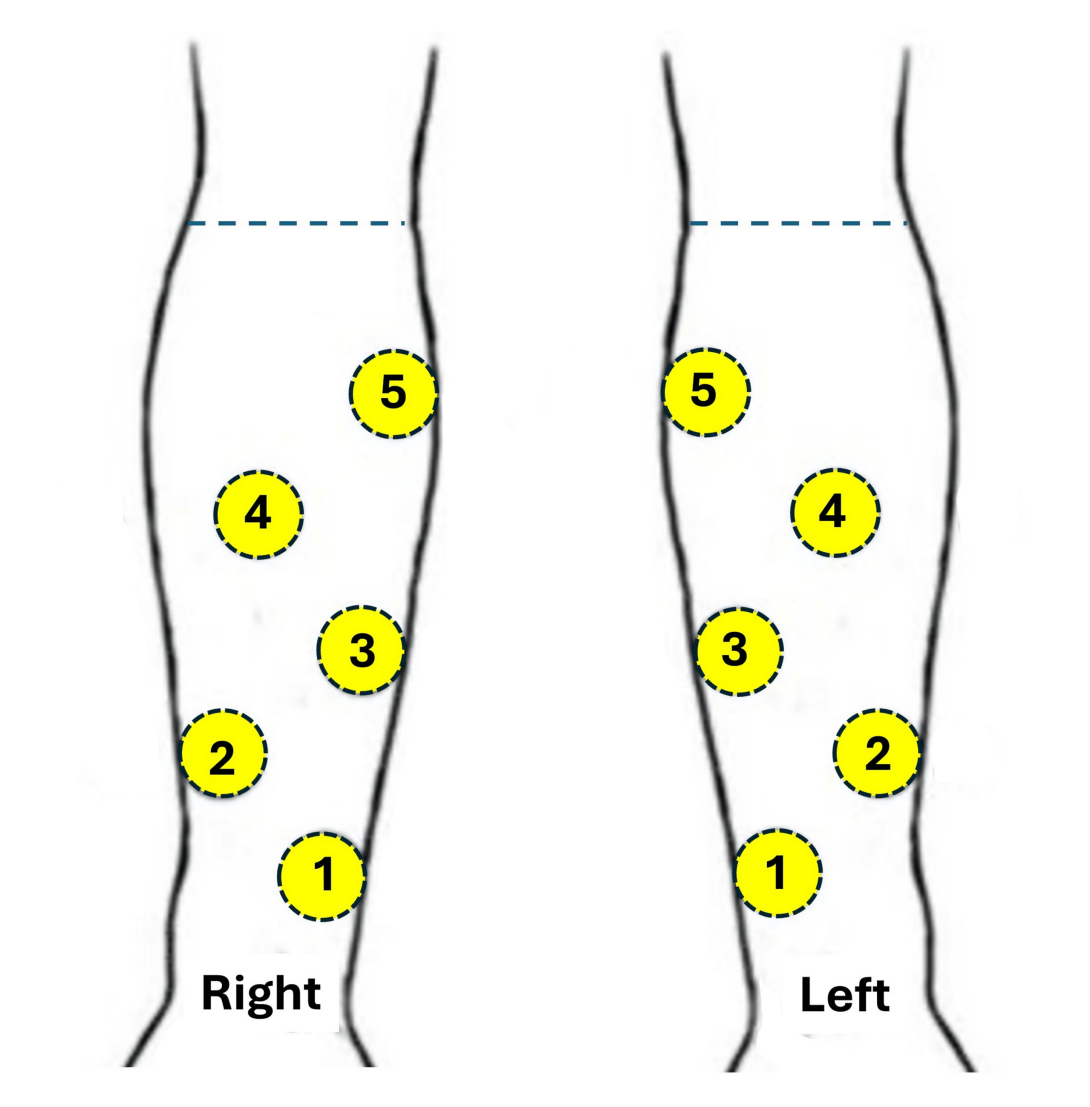
Forearm measured sites Five standardized sites on both anterior forearms were marked along the arm using a plastic template with five holes. Sites 1, 3, and 5 along both inner arms were used to measure the participant's skin melanin index, erythema index, and individual topology angle (ITA). The percentage of skin tissue water content (PWC) was measured in all sites before and four hours after application of the humectant moisturizers. Image created by the authors.

Five standardized sites on both anterior forearms were marked along the arm using a plastic template with five holes. The relative location of these marked areas is illustrated in Figure 1. Sites 1, 3, and 5 along the inner arm of both arms were used to measure the participant's skin melanin index (M), erythema index (E), and ITA. These parameters were measured at these three sites on each arm using the SkinColorCatch device (Delfin Technologies, Kuopio, Finland) [12]. This device is a handheld, self-contained colorimeter that utilizes the CIE Lab* color space, an internationally recognized standard for color measurement. L* represents lightness from black to white (0-100), a* represents the green-red spectrum, and b* represents the blue-yellow spectrum. These parameters defined the participant's intrinsic skin color parameter values. Skin water percentages at all sites were determined by measuring the TDC to an effective depth of 0.5 mm using the MoistureMeterDEpi device (MDE1022, Delfin Technologies, Kuopio, Finland) and to an effective depth of 2.5 mm using the LymphScanner device (MDL2252, Delfin Technologies, Kuopio, Finland) [13]. The TDC value depends on the water concentration, and the measured TDC value is converted into percentage water content (PWC) using the formula $PWC = 1.29 (TDC - 1)$ [14]. The values herein are reported as PWC with PWC5 and PWC25 being the values obtained at measurement depths of 0.5 mm and 2.5 mm, respectively. With the participant seated and their arms resting comfortably on a support surface, TDC values were measured first to a depth of 2.5 mm at all included sites, followed by measurements at these sites to a depth of 0.5 mm. Measurements always started with the left arm at site 1 and proceeded to the right arm after the left arm was measured.

Procedures

After completing the baseline PWC measurements, four different humectant moisturizers were applied to four randomly determined sites on both arms. The topical moisturizers used were Cerave Moisturizing Cream, Cetaphil Moisturizing Cream, Eucerin Advanced Repair Cream, and SkinMedica HA5. One site on each arm did not receive moisturizer. After moisturizer application, the participants were excused and instructed to continue normal activities other than exercise, washing, or rubbing their forearms. When a participant returned four hours later, the TDC measurements were repeated at the same sites and with the same pattern as during the baseline measurements. All subjects received the same dosage and were evaluated initially between 8 and 10 a.m. with a follow-up between 12 and 2 p.m. Room temperatures varied from 22.5°C to 24.4°C, and relative humidity varied from 46% to 53%.

Analysis

Each skin color parameter (M, E, and ITA) was determined as the average obtained from the six measured sites (three on each arm). The baseline PWC overall average value was defined as the average of the eight sites (four on each arm) that would receive moisturizer. The overall average PWC at the same site when remeasured four hours after moisturizer application was used to characterize the four-hour PWC value. Individual responses to a specific humectant moisturizer were not of interest and were not determined. The median value for each color parameter (M, E, and ITA) was determined, and two subgroups were defined as (1) values below the median (N = 15) and (2) values above the median (N = 15). Statistical differences in PWC between these two subgroups at baseline and at four hours were tested with the non-parametric Mann-Whitney U test. Statistical differences in PWC between baseline and four hours were tested using the non-parametric Wilcoxon signed rank test. Statistical significance is required, with a p-value < 0.05. Data provided within the text are expressed as mean ± SD.

## Results

Participant features

Table 1 summarizes the main parameters of the entire participant group, which is divided by gender (16 females and 14 males). Participants could be characterized as a young adult group, with an average age of 24.3 ± 1.6 years, and no significant age differences were observed between female and male participants. The overall body mass index (BMI) of the whole group was 24.5 ± 4.9 kg/m², which, as a class, would be regarded as within the normal weight range (18.5-24.9 kg/m²). However, BMI values did not statistically differ between females and males. Males had significantly higher values for melanin (p = 0.007) and erythema (p = 0.043) and had lower ITA values (p = 0.017). Most participants identified themselves as White (43.3%), with 36.7% identifying as Asian or South Asian. The remaining 20% of participants included those who declared themselves as either Middle Eastern, Hispanic, or Black. The median values for the entire group for the melanin index, erythema index, and ITA were 576.8, 416.5, and 39.7, respectively.

**Table 1:** Participant features Table entries for parameters are the mean values for each parameter with the standard deviation in parentheses. ITA refers to the individual topology angle, while race/ethnicity is as expressed by the participant's self-identification. The p-values for female-male differences are determined via the non-parametric Mann-Whitney U test. The p-value considered significant is p < 0.05. Table entries for the race/ethnicity are the number of subjects reporting that race/ethnicity, together with their percentages in parentheses. Female-male differences in race/ethnicity were not evaluated; hence, no p-values are indicated. ITA: individual topology angle; BMI: body mass index

Variables	All	Female	Male	p-value
Number of participants	30	16	14	-
Age (years)	24.3 (1.6)	24.3 (1.5)	24.2 (1.7)	0.854
BMI (kg/m^2^)	24.5 (4.9)	23.6 (4.3)	25.6 (5.3)	0.257
Melanin index (M)	595 (65)	568 (53)	625 (67)	0.007
Erythema index (E)	415 (22)	407 (21)	424 (22)	0.043
ITA (degrees)	36.2 (18.1)	43.3 (14.3)	28.2 (19.0)	0.017
Race/ethnicity count (N)				
White	13 (43.3)	9 (56.3)	4 (28.6)	-
South Asian	7 (23.3)	2 (12.5)	5 (35.7)	-
Asian	4 (13.1)	1 (6.3)	3 (21.4)	-
Middle East	3 (10.0)	1 (6.3)	2 (14.3)	-
Hispanic	2 (6.7)	2 (12.5)	0 (0.0)	-
Black	1 (3.3)	1 (6.3)	0 (0.0)	-

Skin tone parameter differences between genders

Table 2 separately summarizes the differences in skin tone parameters (melanin, erythema, and ITA) for females and males for each subgroup. Subgroup 1 consists of participants with melanin or erythema values below or ITA values above the median value. Subgroup 2 consists of participants with melanin or erythema values above or ITA values below the median. For each skin tone parameter, the male subgroups were significantly different than the females. The males had greater melanin and erythema values and lower ITA values, all pointing to an overall darker skin tone. Differences in male skin compared to female skin, including its thickness, a darker tone due to melanin amounts, and its higher red tone value due to blood vessel effects, have been previously reported [15].

**Table 2:** Skin tone parameters * denotes significantly different from the corresponding female subgroup value, p < 0.05. ** denotes significantly different from the corresponding female subgroup value, p < 0.01. Table entries are the mean and SD in parentheses for each parameter. Subgroup 1 consists of participants with melanin or erythema values below or ITA values above the median value. Subgroup 2 consists of participants with melanin or erythema values above or ITA values below the median. The p-value compares subgroup 1 vs. 2 using the nonparametric Mann-Whitney U test. ITA: individual topology angle; SD: standard deviation

Parameters	Female (N = 8/Subgroup)			Male (N = 7/Subgroup)		
	Subgroup 1	Subgroup 2	p-value	Subgroup 1	Subgroup 2	p-value
Melanin	553.6 (17.1)	602.8 (52.8)	0.0001	572.8** (23.3)	678.2* (53.1)	0.0005
Erythema	391.1 (15.9)	423.3 (9.7)	0.0001	407.2** (14.7)	440.7* (12.9)	0.0005
ITA	53.7 (6.7)	32.9 (12.2)	0.0001	43.0** (8.6)	13.4** (14.0)	0.0005

Baseline percentage water comparisons

Table 3 summarizes PWC values measured to a 0.5 mm depth (PWC5) and a 2.5 mm depth (PWC25) for each melanin, erythema, and ITA subgroup before humectant application (T = 0) and four hours after application (T = 4). Considering the whole group (N = 30), baseline PWC values measured to a 0.5 mm depth were significantly greater than those measured to a 2.5 mm depth (46.4 ± 10.2 vs. 39.1 ± 6.6, p < 0.001). However, for both measurement depths, PWC values were similar for each subgroup, with no statistically significant subgroup differences for melanin index, erythema index, or ITA values.

**Table 3:** PWC values for each subgroup Table entries are the mean and SD in parentheses. PWC5 and PWC25 are percentages of water at depths of 0.5 mm and 2.5 mm, respectively. M1 and M2 correspond to subgroups below (M1) and above (M2) the median melanin index value. E1 and E2 correspond to subgroups below (E1) and above (E2) the median erythema index value. ITA1 and ITA2 correspond to subgroups below (ITA1) and above (ITA2) the median ITA value. T = 0 corresponds to the baseline (pre-humectant application). T = 4 corresponds to four hours after application. PT represents the p-value differences between T = 0 and T = 4 based on non-parametric Wilcoxon signed rank tests. P_X_ represents the p-values for differences between lower and upper median subgroups (1 vs. 2) determined by non-parametric Mann-Whitney U tests. The % PWC is the percent increase from baseline to four hours. A p-value < 0.05 is taken as statistically significant. PWC: percentage water content

PWC5	Melanin Index Groups			Erythema Index Groups			Individual Typology Angle (ITA) Groups		
	M1	M2	P_X_	E1	E2	P_X_	ITA1	ITA2	P_X_
PWC5 at T = 0	46.5 (9.7)	46.3 (11.1)	0.806	44.0 (8.6)	48.7 (11.6)	0.305	47.8 (11.9)	46.4 (10.3)	0.744
PWC5 at T = 4	52.9 (9.5)	50.1 (8.9)	0.367	50.7 (8.3)	52.3 (10.1)	0.902	50.8 (9.3)	51.5 (9.1)	0.595
P_T_	0.001	0.008	-	0.001	0.017	-	0.017	0.001	-
% PWC increase	15.1 (12.0)	10.0 (10.4)	0.367	16.3 (10.1)	8.9 (11.6)	0.089	8.3 (10.6)	16.7 (10.7)	0.081
PWC25	Melanin Index Groups			Erythema Index Groups			Individual Typology Angle Groups		
	M1	M2	P_X_	E1	E2	P_X_	ITA1	ITA2	P_X_
PWC25 at T = 0	39.1 (6.5)	39.1 (7.0)	0.935	37.5 (5.3)	40.7 (7.6)	0.387	40.1 (7.7)	39.1 (6.6)	0.624
PWC25 at T = 4	41.0 (6.5)	40.1 (5.9)	0.595	39.3 (5.3)	41.8 (6.8)	0.539	41.1 (6.7)	40.6 (6.1)	0.983
P_T_	0.002	0.077	-	0.002	0.077	-	0.077	0.002	-
% PWC increase	5.0 (3.9)	3.3 (7.1)	0.074	5.0 (3.7)	3.3 (7.3)	0.061	3.2 (7.1)	5.1 (3.9)	0.056

Four-hour percentage water comparisons

Four hours after humectant moisturizer application, the whole group (N = 30) showed increased PWC at both measured depths. At a depth of 0.5 mm, the mean PWC value increased from its baseline of 46.4 ± 10.2 to 51.5 ± 9.1 (p < 0.001). This represents a 12.6 ± 11.3% increase calculated from the average percentage increase of each participant. At a depth of 2.5 mm, PWC increased from its baseline value of 39.1 ± 6.6 to 40.6 ± 6.1 (p < 0.001), representing an increase of 4.2 ± 5.7%. The subgroup comparisons of Table 2 show that four hours after humectant application, PWC increased for all subgroups (melanin, erythema, and ITA) when measured to a depth of 0.5 mm. However, percentage increases in PWC tended to be greater for subgroups with lower melanin (M1), lower erythema (E1), and higher ITA (ITA2). At a measurement depth of 2.5 mm, the subgroup with a lower melanin index (M1) or a lower erythema index (E1) demonstrated a small but statistically significant increase in PWC. In contrast, the subgroup with high values of these parameters (M2, E2) failed to increase significantly. This pattern is also observed with the ITA values for the subset, where high ITA values correspond to lower melanin values.

Gender comparisons

Table 4 presents the PWC for female and male participants before treatment and four hours after applying humectant moisturizer. Females had lower baseline PWC values at 0.5 mm depth (42.8 ± 7.4) and 2.5 mm depth (35.6 ± 3.95). However, at a depth of 0.5 mm, the percentage increase in PWC at four hours after moisturizer application was similar between females and males. At a depth of 2.5 mm, PWC increases tended to be slightly greater in females but were not statistically significant.

**Table 4:** Gender comparisons Table entries represent the percentage water content (PWC) for female (N = 16) and male (N = 14) participants at baseline and after four hours of humectant moisturizer application. The p-values are based on the non-parametric Wilcoxon signed-rank test. Standard deviations are shown within the parentheses. The % increase is the mean of the individual participants' PWC increase. A p-value < 0.05 is taken as statistically significant.

Depth	Female PWC Values				Male PWC Values			
	Baseline	Four Hours	p-value	% Increase	Baseline	Four Hours	p-value	% Increase
0.5 mm	42.8 (7.40)	48.4 (7.16)	0.0004	13.5 (6.68)	50.4 (11.81)	55.1 (10.0)	0.026	11.5 (15.3)
2.5 mm	35.6 (3.95)	37.1 (3.88)	0.002	4.3 (3.54)	43.1 (6.97)	44.5 (5.90)	0.060	4.1 (7.70)

Within Gender Comparisons

To examine the possible effect of gender per se on moisturizing responses, an analysis was conducted separately for females and males, similar to the analysis on the entire group, which considered subgroups below and above their median melanin value. The median melanin value for females was 553.5, and for males it was 615.75. Table 5 summarizes the outcome of that analysis. At baseline, there were no significant differences in PWC between participants in the lower and higher melanin subgroups. For females, after humectant application, PCW increased at both measurement depths approximately equally for those with low and high melanin levels. A greater percentage increase (13%) was observed at the 0.5 mm depth, with an increase of only 4.1-4.4% at the 2.5 mm depth. Contrastingly, for males, the increase in PWC occurred only among participants in the subgroup with lower melanin levels. Similar patterns were found for lower and upper erythema and ITA values.

**Table 5:** PWC values for each subgroup by gender PWC5 and PWC25 are the percentages of water content (PWC) at depths of 0.5 mm and 2.5 mm, respectively. M1 and M2 correspond to subgroups below (M1) and above (M2) the median melanin index value. T = 0 corresponds to the baseline (pre-humectant application). T = 4 corresponds to four hours after application. PT represents the p-value differences between T = 0 and T = 4 based on non-parametric Wilcoxon signed-rank tests. P_X_ represents the p-values for differences between lower and upper median subgroups (1 vs. 2) determined by non-parametric Mann-Whitney U tests. Values in parentheses are standard deviations, and % PWC is the percent increase from baseline to four hours. A p-value < 0.05 is taken as statistically significant.

PWC5	Female (8/group)			Male (7/group)		
	M1	M2	P_X_	M1	M2	P_X_
PWC5 at T = 0	45.6 (9.25)	40.0 (3.69)	0.234	48.6 (10.3)	52.3 (13.7)	0.620
PWC5 at T = 4	51.5 (9.07)	45.2 (2.24)	0.234	57.3 (8.32)	52.8 (11.7)	0.456
P_T_	0.012	0.012	-	0.028	0.735	0.053
% PWC increase	13.5 (6.62)	13.5 (7.20)	0.999	20.2 (15.0)	2.8 (10.2)	-
PWC25	Female (8/group)			Male (7/group)		
	M1	M2	P_X_	M1	M2	P_X_
PWC25 at T = 0	37.6 (4.68)	33.7 (1.78)	0.065	43.6 (6.63)	42.6 (7.79)	0.949
PWC25 at T = 4	39.1 (4.45)	35.2 (1.99)	0.105	45.6 (6.39)	43.3 (5.69)	0.535
P_T_	0.018	0.018	-	0.028	0.752	-
% PWC increase	4.1 (3.22)	4.4 (4.04)	0.959	4.9 (3.96)	3.1 (10.5)	0.073

## Discussion

The rationale for this research was based on various studies that combined skin tone parameters and skin hydration measurements in individuals of different races, ethnicities, and geographic locations, reporting baseline hydration differences [1,10,16-18]. Our primary goal was to investigate whether melanin, erythema, or skin ITA values, as measures of skin tone, influenced the effectiveness of humectant moisturizers in hydrating the skin. For this purpose, forearm skin PWC was measured before and four hours after application of four different humectant moisturizers, each applied at different forearm sites. The potential influence of each skin tone parameter on skin hydration was evaluated by comparing the PWC responses of participants with skin tone parameters below and above their median value for each parameter. For convenience, the subgroup with melanin or erythema values less than the group median or ITA values greater than the median (characterized by a lighter skin tone) is referred to as subgroup 1, and the others are designated as subgroup 2.

**Figure 2:**
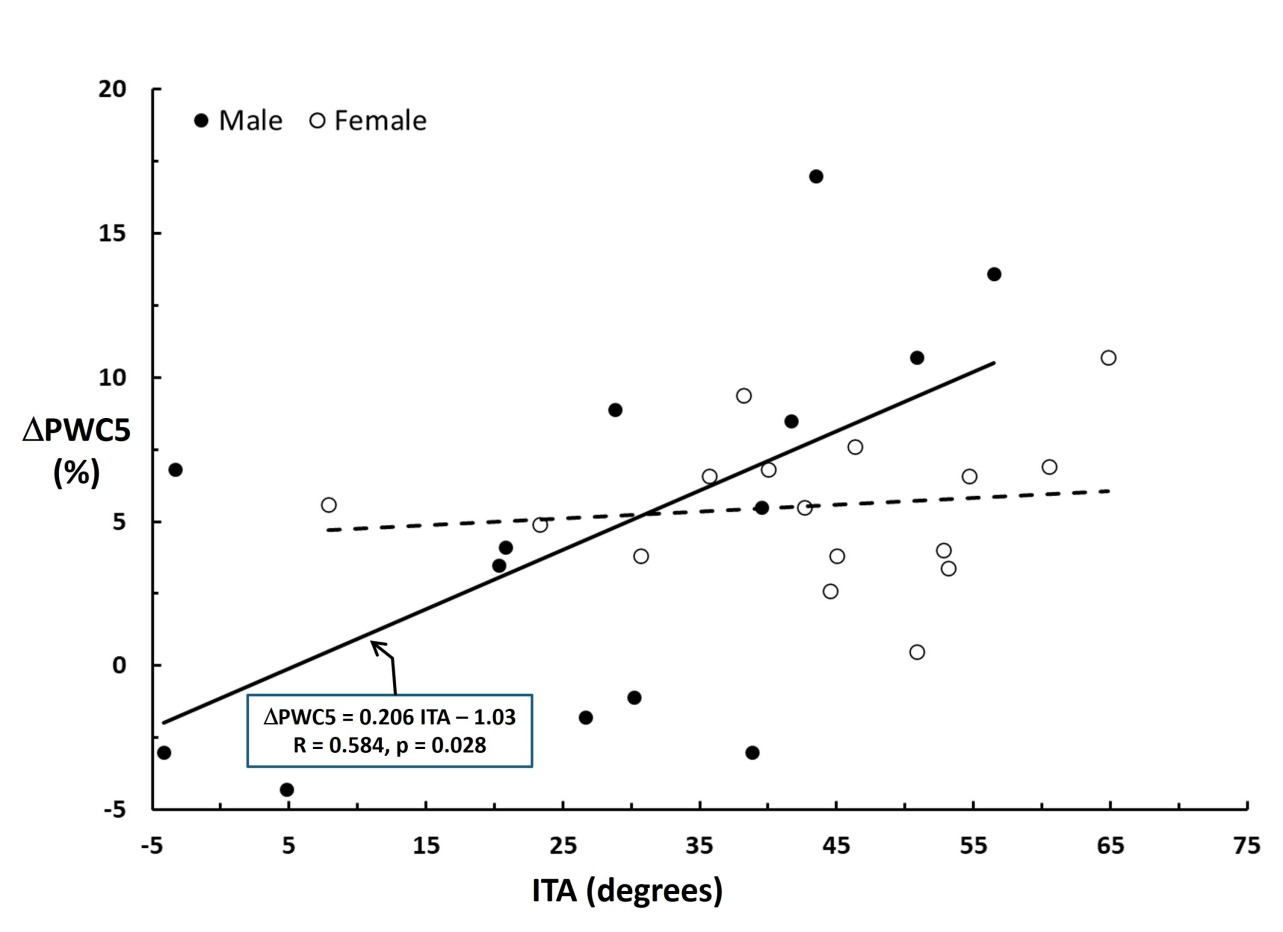
Change in PWC after moisturization Change in PWC measured at a depth of 0.5 mm for males (dark circles) and females (light circles) four hours after moisturizer application vs. the participant's ITA. The solid line is the linear regression for males, and the dashed line is for females. Only the correlation for males is statistically significant based on its p-value being < 0.05. PWC: percentage water content; ITA: individual topology angle

For the entire group of 16 females and 14 males, it was found that when measurements were made to a depth of 0.5 mm, all participants, independent of their subgroup, demonstrated statistically significant increases in PWC due to humectant application. However, the percentage increase tended to be greater for participants in subgroup 1, although the statistical significance of the difference between subgroup 2 was not achieved. The response pattern for measurements to a 2.5 mm depth differed in two ways. The absolute increase was only statistically significant for the participants in subgroup 1, and the percentage increase was slight and generally not statistically significant. These composite findings suggest the following: Firstly, the humectant's largest effect is found at the shallower depth, which at 0.5 mm includes the epidermis and a portion of the dermis. This finding might have been qualitatively inferred but is herein quantitatively expressed. Secondly, and directly bearing on the study's goal, the hydration effect of the humectant tends to be greater for individuals with lighter skin tones, who are characterized by lower melanin or higher ITA values. However, there may be a potential problem with assigning the greater hydration simply due to lighter skin tones without considering a possible gender-related effect. The analysis of the overall response to humectant moisturizer application, separately by gender, showed highly significant increases in PWC for females but less so for males (Table 4), suggesting some intrinsic difference between females and males. To explore this aspect, a subgroup analysis was conducted for females and males separately, comparing responses to high and low melanin subgroups (Table 5). The outcome of that analysis showed essentially no subgroup difference in hydration responses for females with PWC, with both subgroups increasing on average by 13.5%. Contrastingly, for males, PWC increased only for participants in subgroup 1, the subgroup with lighter skin tones. The overall gender-related differential response is visualized in Figure 2, which shows the change in male and female PWC5 (ΔPWC5) calculated as the PWC5 value at four hours minus the baseline value.

For males, there is a moderate positive correlation between the change in PWC5 and ITA (R = 0.584, p = 0.028), but no significant correlation (R = 0.133, p = 0.623) between the change in PWC5 and ITA was present for females. This can be interpreted as an improved hydration response for males with lighter skin tones compared to the more uniform response displayed by females. It is unclear whether this finding was due to intrinsic differences between genders or other factors. Although some studies have reported on hydration levels in different ethnic populations with varying skin tone parameters [2,17,19], these have primarily been conducted in females. Comparisons of baseline hydration levels in males vs. females in a Chinese population have indicated greater values in males [20] but reported as being less than in females in another study [21]. A systematic review reported no gender differences in stratum corneum hydration [22], with a recent study also finding no baseline gender differences [23]. Differences in male-female skin hydration among races have been described at various anatomical locations [24]. However, the specific aspect of gender-related differences in responses to moisturization has not been previously reported. The results of the present pilot study offer an initial basis for further inquiry into this issue. Furthermore, the results also provide initial baseline and response hydration data linked to quantitative measures of skin tone parameters, potentially valuable in their own right.

Study limitations

The possible gender-related differences observed in the present study suggest that future studies addressing the issue of skin tone's impact on responses to moisturization should include larger numbers of participants within each gender, with a more balanced distribution of skin tones across the range of possible skin tones.

Another limitation is the number of participants and their demographics. The present findings are specific to the young adult healthy population studied herein, and potential generalizations to older populations or individuals with any skin condition must await further investigation. Such studies could use the present results as baseline indicators. Moreover, the initial quantitative data may serve as an initial comparison subset, although this is strictly applicable to young adult health subjects.

Another possible limitation is that the responses to individual products were not analyzed separately, and the reported responses were presented as an aggregate. However, this approach was consistent with the goal of the present study.

## Conclusions

Four hours after applying a combination of humectant moisturizers to the forearm, the skin PWC increased for both females and males. The increase was significantly greater at a 0.5 mm depth compared to a 2.5 mm depth, and it was greater for participants with higher ITA values corresponding to lighter skin tone. This suggests that skin tone values may affect achieved hydration levels. However, when considering the potential role of gender, the hydration response to moisturization was more uniform for females, being less dependent on skin tone parameter values than for males, who had a statistically significant positive correlation between moisturizer-induced skin water increase and ITA values. It is unclear whether this gender difference is due to intrinsic differences between genders or other factors, and these findings should be viewed as exploratory. However, the results of the present study provide initial baseline and response hydration data linked to quantitative measures of skin tone parameters, potentially valuable in their own right and as an initial comparison data set for future studies.
